# HDR Syndrome Accompanying Type 1 Diabetes Mellitus and Hypopituitarism

**DOI:** 10.1155/2019/7276947

**Published:** 2019-05-16

**Authors:** Mustafa Can, Feridun Karakurt, Muhammed Kocabas, İlker Cordan, Melia Karakose, Mustafa Kulaksızoglu

**Affiliations:** Department of Endocrinology and Metabolism, Meram School of Medicine, Necmettin Erbakan University, Konya, Turkey

## Abstract

HDR (Hypoparathyroidism, Deafness, and Renal Dysplasia) syndrome is an autosomal dominant disorder characterized by the triad of hypoparathyroidism, sensorineural deafness, and renal disease. Approximately 65% of patients with HDR syndrome have all three of these features, while others have different combinations of these features. We aimed to present a case with primary hypoparathyroidism, hearing loss, and nondiabetic chronic kidney disease and diagnosed as HDR syndrome while being followed up for type 1 diabetes mellitus and hypopituitarism.

## 1. Introduction

HDR syndrome is an autosomal dominant disorder with a broad clinical spectrum. HDR syndrome is caused by mutations in the GATA3 gene found in the 10p chromosome (10p14) [1–3]. The frequency of the disease is unknown. Patients may present with hypocalcemia, tetany, or convulsion at any age. Other classical features include hearing loss and renal diseases such as nephrotic syndrome, renal dysplasia, hypoplasia or aplasia, pelvicalyceal deformity, vesicoureteral reflux, and chronic renal failure [4–6].

The diagnosis of HDR syndrome is based on clinical findings and can be supported by measurement of parathyroid hormone levels, an audiogram or auditory brainstem response, kidney imaging studies, and kidney biopsy. In addition to classical findings, patients with HDR syndrome may have diseases such as Hirschsprung disease, renal tubular acidosis, autoimmune thyroiditis, and type 1 diabetes mellitus. In this article, we aimed to present a case with primary hypoparathyroidism, hearing loss, and nondiabetic chronic kidney disease and diagnosed as HDR syndrome while being followed up for type 1 diabetes mellitus and hypopituitarism.

## 2. Case Report

A 28-year-old male patient was admitted to endocrinology outpatient clinic with complaints of impaired balance, hearing loss, and numbness in the hands for a long time. There were type 1 diabetes mellitus, hypopituitarism, and short-term myoclonic jerks after head trauma in his medical history. The drugs he used were insulin aspart, insulin glargine, and carbamazepine. In the family history there was a consanguineous marriage between the parents. His family did not have any history of HDR syndrome. Vital signs upon admission found a blood pressure of 100/70 mmHg, heart rate of 84 beats/min, and body temperature of 36.6°C. He was not mentally retarded. In the neurological examination, the patient was awake, oriented, and cooperative with exam; cranial nerves, motor strength, sensory testing, and proprioception were all intact; only ataxic gait was present. Hair in the face and axillary and inguinal regions was decreased, bilateral testes were small, and gynecomastia was present. All other examinations were normal.

In laboratory examination, the following are found: leukocyte: 8.6 10^3^/ml (4-10), hemoglobin: 10.8 gr/dl (12.1-17.2), MCV: 99.7 fL (82.2-99), platelet: 119 10^3^/ml (150-400), glucose: 386 mg/dL (70-100), urea: 80 mg/dL (16.6-48.5), creatinine: 1.9 mg/dL (0.70-1.2), sodium: 161 mmol/L (136-145), potassium: 4.48 mmol/L (3.5-5.1), calcium: 5.68 mg/dL (8.4-10.2), phosphorus: 5.7 mg/dL (2.5-4.5), albumin: 4.02 g/dL (3.5-5.2), HbA1c: 7.6% (4-6), TSH: 1.42 mU/L (0.27-4.2), free T4: 0.546 ng/dL (0.93-1.7), free T3: 1.53 ng/L (2-4.4), iPTH: 19.90 ng/dL (12-88), FSH: 1.3 U/L (1.7 -12), LH: 7.67 IU/L (1.24-8.62), total testosterone: 8.88 ng/dL (249-836), cortisol: 10.15 mcg/dL (6.02-18.4), prolactin: 8.19 mcg/L (4.4- 15.2), B12: 273.2 ng/L (197-771), folic acid: 1.9 mcg/dL (3.89-20), urine analysis Ph: 5.5, density: 1014, microalbumin in spot urine: 0.34 mg/dl (0-2), microprotein in spot urine: 36.3 mg/dl (0-15), creatinine in spot urine: 227.61 (39-259) mg/dl, serum osmolarity: 394 mOsm/KgH2O (285-295), and urine osmolarity: 590 mOsm/KgH2O (50-1200). On abdominal ultrasonography, the size of left kidney was preserved, long axis of right kidney was 8.5 cm, and there was a minimal reduction in cortex thickness and a grade 1 increase in right kidney parenchyma echogenicity. As a result of laboratory and imaging findings, in addition to the diagnosis of type 1 diabetes mellitus and hypopituitarism, chronic kidney disease, primary hypoparathyroidism, and megaloblastic anemia due to folic acid deficiency were detected.

**Figure 1:**
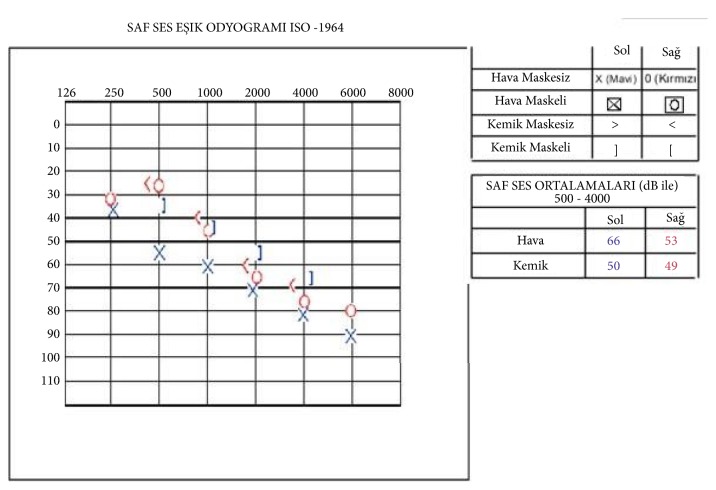
Pure tone threshold audiogram shows a moderate mix type in the left ear and sensorineural hearing loss in the right ear at slightly higher frequencies.

**Figure 2:**
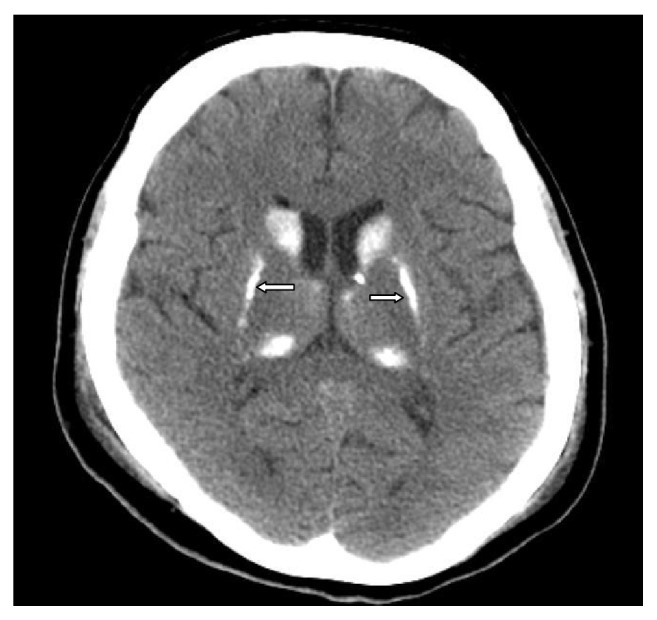
Bilateral symmetrical calcifications are seen in the basal ganglia of the brain CT.

The audiogram showed a moderate mix type sensorineural hearing loss in the left ear and mild sensorineural hearing loss at increased frequencies in the right ear [Figure 1]. There were no pathological findings on eye examination. Bilateral symmetrical calcifications were detected in the basal ganglia, vermis, and cerebellum in brain CT [Figure 2]. There were no pathological findings in the pituitary MRI.

HDR syndrome was considered because of the diagnosis of primary hypoparathyroidism, hearing loss, and nondiabetic chronic kidney disease in addition to type 1 diabetes mellitus, hypopituitarism, and epilepsy. The patient's electrolyte and hormone replacement therapies were held. The symptoms and signs improved and the patient was taken to regular follow-up program.

## 3. Discussion

HDR syndrome is an autosomal dominant disorder. HDR syndrome is caused by mutations in the GATA3 gene found in the 10p chromosome (10p14). The GATA3 gene belongs to the family of double zinc-finger transcription factors involved in the embryonic development of the parathyroid glands, hearing system, kidney, thymus, and central nervous system. The lack of GATA3 mutations and the variability of gene expression lead to phenotypic heterogeneity [1–3].

HDR syndrome can be detected at any age from early childhood to adulthood. The clinical spectrum of HDR syndrome is quite broad and patients may present with clinical findings such as hypocalcemia, tetany, convulsion, hearing loss, nephrotic syndrome, renal dysplasia, hypoplasia or aplasia, vesicoureteral reflux, and chronic renal failure. Ferraris et al. reported that 48 (62.3%) of the 77 patients with HDR syndrome had a complete clinical triad (hypoparathyroidism, sensorineural hearing loss, and kidney disease), 22 (28.6%) had no kidney disease, 2 (2.6%) had no hypoparathyroidism, and 5 (6.5%) had no hearing loss [7].

The diagnosis of HDR syndrome is based on clinical findings and can be supported by measurement of parathyroid hormone levels, an audiogram or auditory brainstem response, kidney imaging studies, and kidney biopsy. Diagnosis is confirmed in patients with HDR triad or patients with two of these three characteristics and a positive family history. In patients with isolated hearing loss or kidney disease who do not meet the above criteria, the GATA3 gene mutation should be detected to confirm the diagnosis [1]. The treatment of patients with HDR syndrome is symptomatic and depends on specific clinical findings and severity of the disease. The prognosis of HDR syndrome is determined by the severity of kidney disease.

Our patient had a complete clinical triad. Therefore, HDR syndrome was diagnosed based on the patient's clinical findings without genetic evaluation. Our case is differentiated from other cases in the literature in terms of accompanying type 1 diabetes mellitus and hypopituitarism.

Muroya et al. reported a case of type 1 diabetes mellitus associated with HDR syndrome. In HDR syndrome, it is thought that GATA 3 haplo insufficiency may cause diabetes mellitus by affecting the function of *β* cells and/or lymphocytes in patients with predisposition due to the genetic and environmental factors [8]. Pituitary insufficiency may occur due to hereditary and acquired disorders. Clinical signs and symptoms vary according to the lacking hormone, degree of deficiency, and the time of onset. The association of HDR syndrome and hypopituitarism has not been reported previously. This association can be coincidental because hypopituitarism was not detected in patients with GATA 3 mutations before.

A total of 108 cases of HDR syndrome have been reported in the literature; some of them are family reports. A total of 4 cases were reported from our country. In addition to classical findings in patients with HDR syndrome, Hirschsprung disease [9], renal tubular acidosis, autoimmune thyroiditis, hypergonadotropic hypogonadism [10], biliary atresia [11], ichthyosis [12], severe mental retardation [13], congenital choanal atresia [14], psoriasis [15], squamous cell lung carcinoma [16], band keratopathy, pigmentary retinopathy [17], nephrocalcinosis [18], cerebral infarction [19], recurrent cerebral infarction [20], tumoral calcinosis [21], and proliferative glomerulonephritis [22] were reported in previous publications.

In this article, we present the case diagnosed with HDR syndrome while being followed up with type 1 diabetes mellitus and hypopituitarism. Further studies are needed to explain the relationship between GATA3 gene and endocrinopathies such as type 1 diabetes mellitus and hypopituitarism.
